# Membrane Repair Mechanisms against Permeabilization by Pore-Forming Toxins

**DOI:** 10.3390/toxins10060234

**Published:** 2018-06-09

**Authors:** Asier Etxaniz, David González-Bullón, César Martín, Helena Ostolaza

**Affiliations:** Biofisika Institute (UPV/EHU, CSIC) and University of the Basque Country (UPV/EHU) Parque Científico s/n, 48940 Leioa, Spain; aetxaniz7@gmail.com (A.E.); david_go88@hotmail.com (D.G.-B.); cesar.martin@ehu.eus (C.M.)

**Keywords:** membrane permeabilization, membrane repair, pore-forming toxins, RTX toxins, adenylate cyclase toxin, *Bordetella pertussis*

## Abstract

Permeabilization of the plasma membrane represents an important threat for any cell, since it compromises its viability by disrupting cell homeostasis. Numerous pathogenic bacteria produce pore-forming toxins that break plasma membrane integrity and cause cell death by colloid-osmotic lysis. Eukaryotic cells, in turn, have developed different ways to cope with the effects of such membrane piercing. Here, we provide a short overview of the general mechanisms currently proposed for plasma membrane repair, focusing more specifically on the cellular responses to membrane permeabilization by pore-forming toxins and presenting new data on the effects and cellular responses to the permeabilization by an RTX (repeats in toxin) toxin, the adenylate cyclase toxin-hemolysin secreted by the whooping cough bacterium *Bordetella pertussis*, which we have studied in the laboratory.

## 1. Introduction

The plasma membrane, a thin layer of only ≈40 Å thickness, regulates the necessary flow of matter, energy, and information between the cell interior and the external medium for cells to live, and thus, its structural integrity is essential for the proper functioning of cells. Membrane permeabilization, independently of the means by which it is caused, may constitute a life or death risk for any cell, and hence, it is expectable that cells possess evolutionarily conserved mechanisms to rapidly repair the injured membranes and ensure survival.

Despite it was reported previously that wounded eukaryotic cells (sea urchin eggs) repair more or less large wounds in their plasma membrane in a few seconds, by a mechanism strictly dependent on extracellular Ca^2+^ [1,2], a systematic study of plasma membrane repair was not developed till the 1990s, with the works of McNeil and Steinhardt [3], who demonstrated that Ca^2+^ entry into wounded cells triggers exocytosis of intracellular vesicles close to the wound site [4,5]. From then, it was clear that plasma membrane repair represents an active, energy-dependent process that is orchestrated by specific membrane traffic events [6]. Here, we provide a short overview of the more general mechanisms currently proposed for plasma membrane repair, focusing more specifically on the cellular responses to membrane permeabilization by pore-forming toxins, and presenting new data on the cellular responses to the pores formed by an RTX (repeats in toxin) toxin, the adenylate cyclase toxin-hemolysin secreted by the whooping cough bacterium *Bordetella pertussis*, which we have studied in the laboratory.

## 2. “Holes” in the Membrane: Some General Aspects

**Figure 1:**
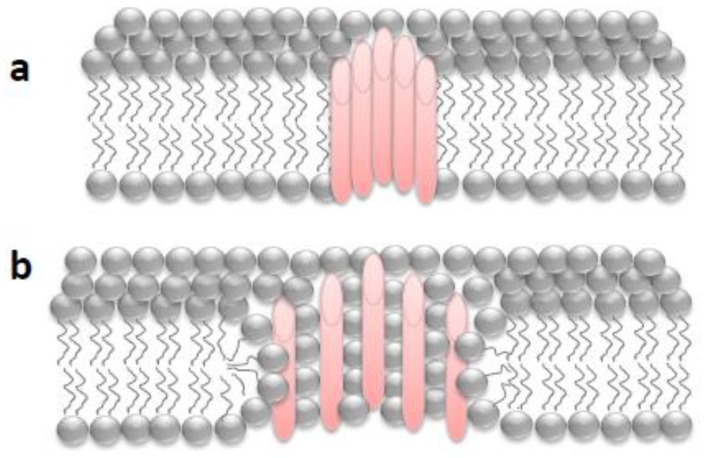
Schematic depiction of different pores with a defined boundary inserted into a biological phospholipid membrane: (**a**) pure proteinaceous pore in which the pore lumen is formed exclusively by protein segments; (**b**) proteolipidic or toroidal pore in which the pore lumen is formed by both lipids and protein segments.

The permeability barrier of the plasma membrane can be frequently breached during the lifetime of most cells by different means: external mechanical forces [7], internal forces generated by contraction and/or migration [8], or pore-forming toxins secreted by pathogens [9,10,11], and thus, the size and nature of the “holes” that can be formed are heterogeneous, and so the repair mechanisms will also vary. Wounds due to mechanical scratching are not delimited by protein boundaries and have an exclusively lipidic lumen (“lipid pores”), while membrane damage by pore-forming proteins give rise to holes with defined boundaries (“proteinaceous or proteolipidic pores”) (Figure 1).

When a simple “lipid pore” forms in the membrane, an energetically unfavorable situation is created, and most likely the hole will tend to seal spontaneously [12]. The anchoring of the membrane to the cytoskeleton and the presence of transmembrane proteins make the membrane more resistant and favor the spontaneous closure of this kind of hole, although this resistance is within damage limits [13] and will in turn be influenced by the lipid composition of the membrane [14]. By contrast, pore-forming proteins create stable holes that do not close spontaneously, and an energy cost paid by cellular machinery will be necessary to close or eliminate the pore [13]. Even in the case of the lipid holes, a drop in membrane tension may not be sufficient to fully close them, and it is likely that active cellular mechanisms are also involved in the closure of most wounds [13].

When studying the cell responses to permeabilization by pore-forming toxins, two important aspects to consider are: the pore size (small pores of less than 1.0 nm in diameter to large oligomeric pores of more than 30 nm) and the secondary structure of the pore formed in the lipid membrane (α-helix/β-barrel), which can determine the repair mechanism that will be activated, or its efficiency, in terms of speed. Furthermore, in the last years, accumulated evidence indicates that certain toxins form the so-called “toroidal pores” [15] in membranes, in which the pore lumen is composed of both proteins (or protein segments) and lipids of different curvature that interact with each other. Toroidal pores differ from the more classical purely proteinaceous pores in that: (i) the pore characteristics depend on the membrane lipid composition, (ii) they have lower stability than pure proteinaceous pores, and (iii) the pore size may vary with the protein concentration and the incubation time [15,16].

## 3. Different Strategies to the Same Problem: Patching, Clogging, Shedding, or Endocytosis

After plasma membrane damage, cells may activate different repair mechanisms, which have been classified in four general strategies, namely: patching, clogging, shedding, and endocytosis [17]. The first two are involved in the repair of damage of mechanical/physical origin, while the last two have been described in the elimination of pore-forming toxins (PFT). In some cell types, different pathways may be complementary and might operate simultaneously [17].

### 3.1. Patching

**Figure 2:**
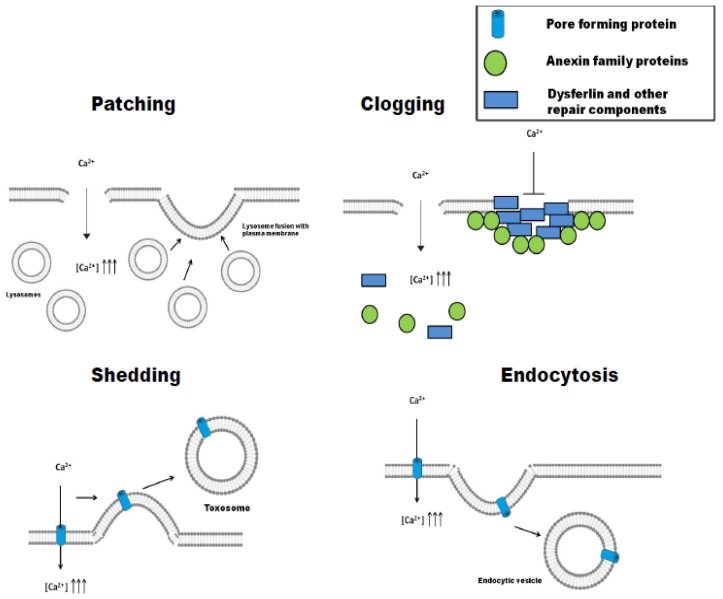
Schematic depiction of different membrane repair pathways: patching, clogging, shedding, and endocytosis. In repair by patching, membrane repair is achieved by fusion of intracellular vesicles with the injured plasma membrane at the wound site, which seals or “patches” the lesion. The mechanism of clogging involves an accumulation of proteins around the lesion, forming a barrier (clog) that prevents the loss of cytoplasmic contents to the extracellular medium, and isolates the area of membrane damage. By the mechanism of shedding, it is possible to isolate and expel the damaged membrane area in the form of vesicles called “toxosomes” or “ectosomes”. Endocytosis-mediated pore removal involves sequential steps of exocytosis and endocytosis. In a first step, lysosomes fuse with the plasma membrane, releasing the lipid hydrolytic enzyme acid sphingomyelinase (ASM) into the extracellular medium, which converts membrane sphingomyelin into ceramide. This lipid seems to create a ceramide platform, which in a second step, induces an invagination of the membrane that promotes its engulfment.

Membrane repair is achieved by fusion of intracellular vesicles with the injured plasma membrane at the wound site, which closes or “patches” the lesion (Figure 2) [7]. Early studies in the 1990s had already noted that membrane damage was repaired with membranes coming from cytoplasmic vesicles [5,18]. The vesicles were mainly lysosomes [19], and the influx of extracellular Ca^2+^ was essential for the activation of this process [4]. As a variant of this mechanism, the so-called “tension release hypothesis” was also proposed [20], according to which, a direct fusion of the internal membranes with the lesion site would not be necessary, but rather the fusion itself would decrease the membrane tension, that would in turn facilitate the closing of the wound. The mechanism of membrane patching seems more appropriate for repairing large “lipid pores” created in the cell membranes by mechanical stresses, but may not be effective against pure “proteinaceous pores” generated by PFTs, since these can be very resistant to changes in membrane tension [3].

### 3.2. Clogging

Plasma membrane repair by this mechanism involves an accumulation of proteins around the lesion, forming a barrier (clog) that prevents the loss of cytoplasmic contents to the extracellular medium and isolates the area of membrane damage [21,22] (Figure 2). Clogging has been implicated in the repair of medium-size membrane lesions (from one to a few microns) [23,24,25]. Although a large variety of proteins putatively involved in this mechanism have been described, the family of annexins seems to be the most important and main regulator of the process. Annexin A1, A2, A4, A5, A6, A7, and A11 have been detected in this process, though annexin A1, A5, and A6 seem to be the most important [17,24,26]. Annexins are activated in response to increases in the intracellular concentration of Ca^2+^ and specifically bind to phosphatidylserines (PS) present in the inner side of the plasma membrane [22]. The different annexins have different calcium sensitivities for their activation and subsequent membrane binding, which confers to the cell the capacity to “sense” the severity and localization of the membrane damage and the possibility of regulating the type of response that will be activated in each particular case [27,28]. As an example, annexin A6 will bind to the membrane upon minor damage, since it is activated at very low calcium concentrations (free [Ca^2+^] ≤ 5 µM), while annexin A1 will only bind when the membrane lesion is more important and involves larger Ca^2+^ entry (free [Ca^2+^] > 10–20 µM) [24,29]. Besides annexins, in different cell types, membrane clogging would involve additional protein partners, such as dysferlin [30], or other repair components such as EHD1, EHD2, MG53, and BIN1, which would establish a complex network of interactions as recruiting platforms [23].

### 3.3. Shedding (Ectocytosis)

By this mechanism, it is possible to isolate and expel the damaged membrane area in the form of vesicles called “toxosomes” or “ectosomes” [31] (Figure 2). Shedding is one of the two mechanisms by which small pores (<100 nm in diameter) are repaired, and it has been described for several pore-forming toxins [31,32,33]. It is directly related to clogging, since the first step is to isolate the damaged membrane area, which forms a bleb that is then pinched off to restore membrane integrity [32]. For the pores formed by the *Staphylococcus aureus* alpha-hemolysin, it has been proposed that shedding occurs after the toxin has been endocytosed and it has not been possible to degrade it, leading to it being expelled from the cell [34]. Proteins of the ESCRT (endosomal sorting complex required for transport) machinery have been directly implicated in the budding and vesicular fission steps required for shedding [17,35,36].

### 3.4. Endocytosis

This is the second mechanism by which small pores formed by PFT are removed from the membrane. In this case, the cell responds by quickly internalizing the damaged area, including the pore [37]. Several PFT and some pore-forming proteins such as perforin [38], *Staphylococcus aureus* α-toxin [34], streptolysin-O [39], and *Vibrio cholerae* cytolysin [40] have been reported to be removed by this repair mechanism. Endocytosis-mediated pore removal involves sequential steps of exocytosis and endocytosis (Figure 2). In a first step, lysosomes fuse with the plasma membrane, releasing their contents to the extracellular medium [39]. Among the released material is the lipid hydrolytic enzyme acid sphingomyelinase (ASM), which converts membrane sphingomyelin into ceramide. This lipid seems to create a ceramide platform, which in a further, second step, induces an invagination of the membrane that promotes its engulfment [39,41]. In fibroblasts, the endocytosis-mediated membrane repair has been localized to caveolae [42], though it is possible that in cells that do not express caveolin, such as certain immune cells, endocytosis may be coupled to clathrin [43]. Cells appear to have a “pool” of lysosomes in the vicinity of the membrane that can fuse quickly with it [44]. The process is triggered by the influx of extracellular Ca^2+^ [19], and it has been postulated that calcium-sensitive proteins in the lysosome membrane might “guide” these vesicles to the injured area [45]. Other released calcium-dependent cysteine proteases such as cathepsins B, D, and L have been implicated in autoregulation of the process [46], avoiding excessive damage. The ESCRT machinery is also involved in the endocytosis-mediated pore removal, together with several RAB proteins (Rab-5 and Rab-11) [9,47]. This exocytosis/endocytosis-based repair mechanism is energy-dependent and requires ATP, besides Ca^2+^, to restore the membrane integrity [37].

## 4. Repair Mechanisms Activated by Pore-Forming Toxins

From numerous studies, it has been concluded that the size of the pore (lumen diameter) is one of the most crucial factors determining the repair mechanism that will be activated by a PFT to restore homeostasis [48,49]. To have an idea of the size of the pores formed by the different PFTs, they have been classified as “large pores” with diameters above 3.0 nm and that can be as large as 30–40 nm, and “small pores” which show pore diameters below 2.0–3.0 nm. We will go over several examples of repair mechanisms activated by these two groups of pore-forming toxins.

### 4.1. Repair of Large Pores

Toxins of the CDC (cholesterol-dependent cytolysins) family are known to form big transmembrane holes that can exceed 30–40 nm in diameter [50]. The consequences of opening such big “holes” in the membrane could be so deleterious that cells will expectedly activate rapid repair responses that may be similar to the processes followed for membrane ruptures due to mechanical damage. In the last years, the repair mechanisms activated by several of these toxins have been reported: listeriolysin [51,52,53], perfringolysin, and intermedilysin [54], though streptolysin-O has been the most studied [37]. Both shedding- and endocytosis-mediated pore removal have been described in cells attacked by these toxins, though it seems that depending on the cell type, the toxin concentration, and the incubation time, one or the other mechanism might predominate [17]. The extent of the Ca^2+^ influx induced by the toxin seems to make the difference, and so, when the intracellular [Ca^2+^] concentration increase is small, the mechanism of vesicle shedding via annexins would predominate, while at high cation concentrations, toxin endocytosis is detected by the presence of ceramide platforms [24], though some studies have also noted caveolae-mediated entry of the toxins [42]. From the different studies with cholesterol-dependent cytolysins (CDC), it is concluded that repair of these large pores by either of the two mechanisms, shedding or endocytosis, is a rapid process (time scale of seconds to a few minutes) that requires Ca^2+^ and ATP.

### 4.2. Repair of Small Pores

The best known two representatives of toxins forming small pores are α-hemolysin from *Staphylococcus aureus* [33] and the *Aeromonas hydrophila* aerolysin [48]. Both toxins form heptameric transmembrane β-barrel pores of small size (<1.5 nm internal diameter) in target membranes [55]. Pore formation by these toxins leads to the deregulation of ionic homeostasis and a decrease of cellular ATP [48,56]. Relative to the large pores aforementioned, the kinetics of membrane repair for the small pores is much slower, requiring several hours for K^+^ and ATP to return to normal values [48]. Though these toxins can be endocytosed or expelled, the internalization seems less effective, since a considerable amount of the toxin remains in the membrane hours after incubation [45,56]. Extracellular Ca^2+^ is not likely required for repair of small pores formed by *S. aureus* α toxin, as these are Ca^2+^-impermeable [33,57]; however, intracellular Ca^2+^ stores may still play a role. Some authors have proposed that after the toxin has been endocytosed, cells tend to expel them as toxosomes, due to their incapability to be degraded intracellullarly [34]. Cry5B, another toxin that forms small pores of about 1 to 2 nm, was found to activate a repair response in which RAB-5 and -11 and the ESCRT machinery are required [9]. Recent studies with other two toxins, *Photobacterium damselae* Phobalysin (PhPly) and *Vibrio cholerae* cytolysin (VCC), have revealed that even subtle differences in the pore width can be determinant for the type of response that will be activated [49]: the PhPly pore is ≈1.2–3 nm [58], while the VCC pore is slightly smaller ≈1.2 nm [59], but the repair mechanisms activated in each case are different. PhPly activates a lysosome-mediated fast endocytosis, while VCC repair has slow kinetics, similar to those described for α-toxin and aerolysin [48], most likely because the VCC pore is not Ca^2+^-permeable. Mutations in the VCC protein that enlarge the pore size lead to the activation of the lysosome-mediated endocytosis-based repair mechanism [49].

Besides the direct physical elimination of the toxin pores from the permeabilized membranes, several signaling routes are also activated in the perforated cells [60]. K^+^ efflux, more than that of Ca^2+^, seems to be the key signal for activation of these pathways, and the MAPK p38 seems to be the main regulator [61]. Although the effector proteins of the routes are not known, it seems that their participation is crucial for the survival of the cells permeabilized by the Ca^2+^-impermeable pores [48]. In fibroblasts, activation of caspase-1 has been reported to help in cell survival upon the action of small-pore PFTs [62], which highlights the importance of the cell type in the study of signaling pathways, since activation of caspase-1 in immune cells leads to rapid cell death by pyroptosis.

## 5. ACT Toxin: An Adenylate Cyclase Enzyme Fused to an RTX Hemolysin

**Figure 3:**
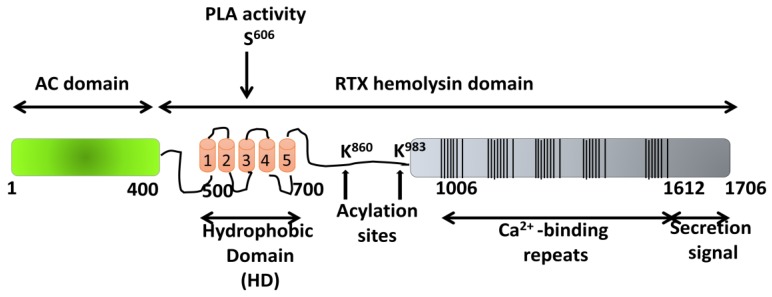
Structural organization of the ACT toxin. The adenylate cyclase domain (AC domain) (in green) extends approximately from residues 1 to 400. The RTX hemolysin domain (residues from ≈500 to 1706) contains the hydrophobic domain (HD) constituted by five hydrophobic/amphipathic alpha-helices (in red); two conserved acylation sites, K860 and K983, required for activation by palmitoylation mediated by CyaC acyltransferase; and five blocks formed by low-affinity calcium-binding repeats. The Ca^2+^-binding region (residues 1006–1612) is denoted by multiple lines, with each line corresponding to single nonapeptide repeats with the consensus sequence GGXGXDXLX. The segment between residues 1638–1706 corresponds to the C-terminal secretion signal. Location of the catalytic residue serine 606 (S^606^), involved in the intrinsic phospholipase A (PLA) activity of the toxin, which has been recently reported by González-Bullón et al. [73], has been included.

Adenylate cyclase toxin (ACT or CyaA) secreted by *Bordetella pertussis*, the bacterium causative of whooping cough (pertussis), has a critical role in the early stages of respiratory tract colonization by this pathogenic bacterium [63]. ACT is a prototypic toxin of the so-called RTX family of proteins characterized by possessing, in their C-terminal sequence, a variable number of glycine- and aspartate-rich repeats of nine amino acids (aa), mostly exhibiting cytotoxic/cytolytic pore-forming activity [64]. ACT is a 1706-residue-long protein, in which an adenylate cyclase (AC) domain of ~400 N-terminal residues is fused to a ~1300-residue C-terminal RTX hemolysin domain (Figure 3). The RTX domain consists of a hydrophobic pore-forming domain (aa ≈ 500–700); an acylation domain, where the post-translational palmitoylation of lysine residues 860 and 983 occurs [65]; a CD11b/CD18 receptor-binding domain (aa 1166–1281) [66]; and an RTX calcium-binding domain harboring the nonapeptide repeats of a consensus sequence X–(L/I/F)–X–G–G–X–G–(N7D)–D, which form numerous (~40) calcium-binding sites. The toxin segment extending approximately from residue 400 to 500, that connects the catalytic AC domain to the pore-forming RTX hemolysin domain, has been recently revealed as possibly being involved in translocation of the AC domain across the cell membrane, assisting membrane insertion [67,68]. All ACT activities depend on the covalent fatty acylation of pro-ACT and on the binding of Ca^2+^ ions to the numerous sites formed in the RTX domain by the glycine- and aspartate-rich repetitions [69]. The toxin targets CD11b/CD18-expressing (αM β2 integrin or CR3) myeloid phagocytes and translocates its adenylate cyclase domain directly across their cytoplasmic membrane without the participation of a receptor-mediated endocytosis step. In the target cytosol, the AC domain is activated by calmodulin [70], acquiring high catalytic activity in the conversion of ATP to cAMP, a key second messenger. The supraphysiological levels of cAMP subvert the signaling of protein kinase A (PKA) and ablate the bactericidal functions of phagocytes, such as oxidative burst and phagocytosis [71]. In parallel, ACT acts as a cytolysin, forming cation-selective pores, which permeabilize the cell membrane and eventually provoke cell lysis. Translocation of the AC domain and oligomerization into cation-selective pores appear to represent two independent and parallel/competing activities of the membrane-inserted form of ACT [72].

### 5.1. Pore-Forming Activity of ACT

Pore formation by ACT has been attributed to the insertion of the hydrophobic/amphipathic α-helical segments located between residues 500 and 700 of the ACT hydrophobic domain (Figure 3) [74,75]. By analogy with known pore-forming toxins, it may be assumed that the process that ultimately leads to osmotic cell lysis by ACT pores consists of three stages: binding, insertion, and oligomerization of the toxin within the membrane. However, because of the lack of high-resolution structures for full-length ACT, or indeed for any of the RTX toxins, knowledge of the lytic mechanism and pore characteristics of these proteins is very limited.

The fact that ACT forms pores in membranes was concluded from conductance measurements in black lipid membranes and hemolysis assays with osmotic protectants [76,77,78]. ACT pores in lipid bilayers were described as transient cation-selective “channels” with an extremely small single-channel conductance of 27 pS in 1 M KCl and a half-life of ~2 s [76]. From those studies, it was estimated that ACT forms a 0.6–0.8-nm pore. More recently, using blue native polyacrylamide gel electrophoresis (BN-PAGE) and immunolabelling, putative ACT oligomers with apparent molecular masses of 200, 300, 410, and 470 kDa have been described in erythrocyte membranes, being attributed to cleaved monomers lacking the AC domain (~200 kDa), full-length monomers (~300 kDa), and ACT oligomers (~410 and 470 kDa), respectively [79]. 

Against the view that RTX toxins form discrete-size pure proteinaceous pores in membranes, additional studies, including our own data, have revealed that the RTX-induced perturbation in membranes depends on a number of factors, including membrane lipid composition, temperature, time, and toxin concentration [80,81]. This suggests that rather than being a static process, permeabilization by RTX toxins may be a complex, dynamic process involving membrane remodeling processes, accompanied by the transient formation of nonlamellar proteolipidic structures in the membrane [80,81,82,83].

### 5.2. Irreversible Membrane Permeabilization by ACT

**Figure 4:**
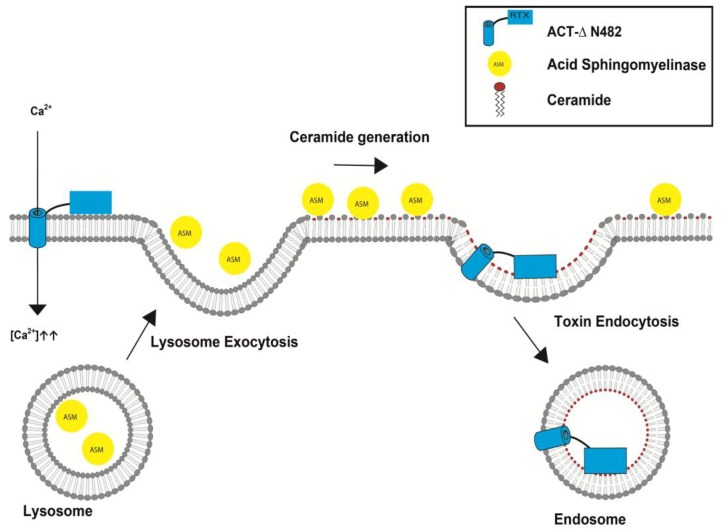
Scheme proposed for the membrane repair pathway activated by the ACT-∆N482 hemolysin pore in macrophages. The ACT-∆N482 hemolysin pore (red) allows for the influx of Ca^2+^ into the cytosol, which is followed by the fusion of lysosomes with the plasma membrane. Fused lysosomes release acid sphingomyelinase (ASM) into the extracellular space. ASM converts sphingomyelin in the membrane to ceramide, generating a ceramide-rich platform which may favor membrane deformation for invagination and subsequent endocytosis of the toxin-ridden membrane into the cell.

Recently, we explored the permeabilization elicited by sublytic doses of ACT on target macrophages and evaluated whether the permeabilized cell membrane was resealed or not, and we found that the full-length toxin induces an irreversible membrane permeabilization that eventually leads to cell death [84] (Etxaniz et al., manuscript in revision), which suggests that cellular repair mechanisms are not operative in the ACT-treated macrophages. Interestingly, an ACT mutant extending from amino acids 483 to 1706 (ACT-∆N482 hemolysin), which contains the entire ACT RTX hemolysin domain, induces by contrast a transient membrane permeabilization that is rapidly reverted in a time scale of minutes (≈30 min) by a repair pathway activated by extracellular Ca^2+^ influx and that requires ATP [84] (Etxaniz et al., manuscript in revision). The increase of intracellular Ca^2+^ induced by the sublytic doses of ACT was found to be very limited [84] (Etxaniz et al., manuscript in revision), and translocation of the adenylate cyclase domain into the cytosol of the macrophages consumes cellular ATP; both factors might contribute to the incapability of the ACT-treated cells to reseal the injured membrane [84] (Etxaniz et al., manuscript in revision). Furthermore, we have found that the repair pathway acting in the ACT-∆N482 hemolysin-treated cells involves consecutive steps of exocytosis and endocytosis, likely initiated by lysosomal fusion with the damaged cell membrane, subsequent secretion to the extracellular medium of acid sphingomyelinase, and concomitant local generation of ceramide, all of which culminates in the endocytosis of the pore-ridden membrane (Figure 4) [84] (Etxaniz et al., manuscript in revision). Given the high similarity among the ACT RTX-hemolysin moiety and the other toxins from the RTX family, it is enticing to surmise that a similar, Ca^2+^-dependent, membrane repair mechanism might also operate for those pore-forming toxins. Up to now, such a membrane repair mechanism had only been reported for toxins forming large beta-barrel pores in membranes, for instance, streptolysin, perfringolysin, pneumolysin, and listeriolysin [51,52,53].

## 6. Conclusions

Membrane repair mechanisms are among the most essential mechanisms to guarantee cell survival. Although the variability of possible repair responses for a given type of lesion may seem striking, a conclusion that can be drawn from all the known examples is that cells are prepared to cope with membrane disruption, triggering a more or less generalized response using all the available resources to resist damage and ensure cell homeostasis. Understanding the mechanism of action of PFT as well as the host responses to toxin action would provide ways to deal with these pathogens or with emerging pathogens, and more importantly, to improve the action of toxins that have biotechnological applications.
